# Decreased GPIHBP1 protein levels in visceral adipose tissue partly underlie the hypertriglyceridemic phenotype in insulin resistance

**DOI:** 10.1371/journal.pone.0205858

**Published:** 2018-11-08

**Authors:** R. Preethi Surendran, Shanti D. Udayyapan, Mercedes Clemente-Postigo, Stefan R. Havik, Alinda W. M. Schimmel, Fransisco Tinahones, Max Nieuwdorp, Geesje M. Dallinga-Thie

**Affiliations:** 1 Department of Experimental Vascular Medicine, Amsterdam University Medical Center, location AMC, Amsterdam, The Netherlands; 2 Unidad de Gestión Clínica Endocrinología y Nutrición, Instituto de Investigación Biomédica de Málaga (IBIMA), Complejo Hospitalario de Málaga (Virgen de la Victoria)/Universidad de Malaga, Malaga, Spain; 3 CIBER Fisiopatologia de la Obesidad y Nutrición (CB06/03), Barcelona, Spain; 4 Department of Vascular Medicine, Amsterdam University Medical Center, location AMC, Amsterdam, The Netherlands; University of Milano, ITALY

## Abstract

GPIHBP1 is a protein localized at the endothelial cell surface that facilitates triglyceride (TG) lipolysis by binding lipoprotein lipase (LPL). Whether Glycosyl Phosphatidyl Inositol high density lipoprotein binding protein 1 (GPIHBP1) function is impaired and may underlie the hyperTG phenotype observed in type 2 diabetes is not yet established. To elucidate the mechanism underlying impaired TG homeostasis in insulin resistance state we studied the effect of insulin on GPIHBP1 protein expression in human microvascular endothelial cells (HMVEC) under flow conditions. Next, we assessed visceral adipose tissue GPIHBP1 protein expression in type 2 diabetes *Lepr* ^*db/db*^ mouse model as well as in subjects with ranging levels of insulin resistance. We report that insulin reduces the expression of GPIHBP1 protein in HMVECs. Furthermore, GPIHBP1 protein expression in visceral adipose tissue in *Lepr* ^*db/db*^ mice is significantly reduced as is the active monomeric form of GPIHBP1 as compared to *Lepr*^*db/m*^ mice. A similar decrease in GPIHBP1 protein was observed in subjects with increased body weight. GPIHBP1 protein expression was negatively associated with insulin and HOMA-IR.

In conclusion, our data suggest that decreased GPIHBP1 availability in insulin resistant state may hamper peripheral lipolysis capacity.

## Introduction

Patients with the Metabolic Syndrome or type 2 diabetes (T2DM) are frequently characterized with atherogenic dyslipidemia, displayed by elevated plasma triglyceride (TG)-rich remnant particles (TRL), associated with increased risk for cardiovascular diseases [1, 2]. However, the underlying pathobiology is still under investigation [1–5]. The first symptoms of elevated plasma TG levels already develops in the early pre-diabetic state when insulin resistance commences [6].

TG are derived from either dietary origin or de novo lipogenesis. Dietary fats are hydrolysed by pancreas lipase in the ileum enabling the uptake of fatty acids in the enterocyte, where they are subsequently re-esterified into TG. TG will be packaged into apoB48-containing chylomicron particles that are secreted into the lymphatic system before entering the systemic circulation [7]. On the other hand, de novo lipogenesis occurs in the liver leading to production of TG that are secreted into apoB100-containing VLDL particles. Abnormalities in TG homeostasis can thus occur through either increased hepatic VLDL secretion or via decreased TRL clearance.

In this respect, lipoprotein lipase (LPL)-mediated lipolysis is the rate-limiting first step to hydrolyse TG in TRL allowing the uptake of free fatty acids (FFA) in peripheral tissues (adipose tissue, heart and skeletal muscle) for storage and energy [8]. LPL is synthesized in the parenchymal cells as a monomeric protein. Lipase maturation factor 1 (LMF1) is required to stabilize LPL by forming the LPL dimer which allows secretion followed by transfer to the endothelial cell surface [9]. It was earlier recognized that the heparin sulfate proteoglycans (HSPGs) facilitate the transcytosis of the secreted LPL by the adipocytes or myocytes to the luminal side of the capillary endothelial cells [10]. In 2008 glycosyl-phosphatidyl-inositol anchored HDL binding protein 1 (GPIHBP1) was identified as the platform to anchor LPL at the cell surface and allow TG hydrolysis to occur [11]. GPIHBP1 is highly expressed in the endothelial cells of adipose tissue, heart and moderately expressed in skeletal muscle, which shows high similarity to the tissue expression profiles of LPL [12]. GPIHBP1 stabilizes LPL by preventing its degradation by ANGPTL4 [13]. LPL is mislocalized in the tissues in the absence of GPIHBP1 and cannot function properly [14]. Consequently, it was recently recognized that GPIHBP1-bound LPL is the main determinant of TRL particle margination in the microvascular circulation [15]. We previously showed the importance of GPIHBP1 in TG homeostasis is illustrated by the identification of patients with severe hypertriglyceridemia who have a homozygous loss-of-function mutation in *GPIHBP1* [16–18].

Mouse models mimicking human type 2 diabetes (T2DM) are characterized by elevated plasma TG levels due to increased production and impaired clearance capacity as illustrated by impaired clearance of postprandial TG [19]. In line, subjects with the Metabolic Syndrome may have insulin resistance which coincides with higher plasma levels of TG. However, it is currently known whether *GPIHBP1* expression in adipose tissue is differentially expressed in an insulin resistance state. Thus, to further understand the mechanism underlying the hyperTG phenotype we evaluated adipose tissue *GPIHBP1* expression in a T2DM mouse model as well as in subjects with ranging levels of insulin resistance. Moreover, we studied the effect of insulin on *GPIHBP1* expression in human microvascular endothelial cells under flow conditions. Here we report that insulin decreases *GPIHBP1* protein expression in endothelial cells *in vitro*. GPIHBP1 protein in visceral adipose tissue is significantly decreased in an insulin resistant mouse model and in subjects with increased body weight. Also, GPIHBP1 protein was negatively associated with insulin and HOMA-IR, suggesting that decreased GPIHBP1 protein availability may hamper peripheral lipolysis and affect plasma TG levels.

## Materials and methods

### Subjects

The study was approved by Institutional Review Board of the Academic Medical Center of the University of Amsterdam (The Netherlands) and carried out according to the Declaration of Helsinki. All participants gave their written informed consent for participation in the study.

The cohort consisted of 45 subjects who were scheduled for elective laparoscopic cholestectomy. Subjects were excluded from participation if they had a history of diabetes mellitus, generalized inflammation or known malignancies. After an overnight fast, blood samples were drawn from a peripheral vein into EDTA-containing tubes and immediately placed on ice. Plasma was separated within 30 minutes by centrifugation at 1,700g for 20 min at 4°C and stored at -80°C for further analysis. Plasma lipid profiles were measured using commercially available assays (Randox, USA and Daiichi, Japan) on a Cobas Mira autoanalyzer (Horiba, France). Plasma glucose, insulin and HbA1c were assessed using standard laboratory procedures. HOMA-IR was calculated using Homeostasis model assessment-estimated insulin resistance (HOMA-IR) as fasting plasma insulin (μIU/ml) x fasting plasma glucose (mmol/l) divided by 22.5. From all participants, a visceral (VAT) adipose tissue biopsy was obtained during the operational procedure, immediately snap-frozen and stored at -80°C for mRNA and protein isolation.

### Animal experiments

All animal experiments were conducted at the Animal Facility of the AMC and approved by the Committee for Animal Welfare (DLV102141-1) of the AMC, Amsterdam, The Netherlands. Eleven-week old male *Lepr*^*db/db*^ mice and control *Lepr*^*db/m*^ mice from the same colony on the C57B/6J background were purchased from The Jackson Laboratory (Bar Harbor, USA). Animals were housed in a constant 12h light/dark cycle with controlled temperature and humidity and were given *ad libitum* access to water and food (Purina lab Diet #5008). At twelve weeks of age, the nonfasting animals were sacrificed and blood samples were obtained. Plasma was isolated by centrifugation for 15 minutes at 3,000 rpm at 4°C and plasma was stored at -80°C for future analysis. Plasma cholesterol and TG were measured with a colorimetric assay (Roche, Basel, Switzerland). Plasma glucose was analysed using strips from Contour (Bayer, Zurich, Switzerland) according to the manufacturers protocol and plasma insulin levels were measured using a commercially available ultrasensitive Elisa from Mercodia (Uppsala, Sweden). Tissues were snap-frozen into liquid nitrogen.

### Protein quantification

Human and mice tissue (~70mg) were homogenized in RIPA buffer containing protease inhibitor cocktail (Roche) and phosphatase inhibitor cocktail (Roche). 25 μg of total protein were size-fractionated on 4–12% polyacrylamide Bis-Tris SDS gels and transferred to PVDF membrane using the Transblot turbo system (BioRad). The membrane was blocked with odyssey block buffer (Westburg) for 1h at room T. The membrane was then probed overnight at 4°C with goat anti-human GPIHBP1 (1:200, sc-87809, Santa Cruz) and mouse anti-human β-actin (1:5000, ab-8226, Abcam) as primary antibodies. The blots were then incubated with donkey anti-goat 800CW IRdye (925–32214, Westburg) and donkey anti-mouse 680RD (925–68072, Westburg). Finally, the blots were imaged using Odyssey Infrared imaging system (LI-COR) and the band intensities were quantified using Odyssey software version 3.0.16 (LI-COR) and expressed relative to β-actin. For measurements of multimer-monomer GPIHBP1 the samples were tested in a non-reducing condition.

### Effect of insulin on GPIHBP1 expression in HMVEC under laminar flow

Human microvascular endothelial cells adult dermis (HMVEC-ad, C-011-5C, Life Technologies,) were cultured in EGM-2MV (Lonza) supplemented with 5%FBS (cc-4102B), 0.2 ml corticosone (cc-4112B), 2 ml hFGF (cc-4113B), 0.5 ml VEGF (cc-4114B), 0.5 ml RB-IGF1 (cc-4115B), 0.5 ml ascorbic acid (cc-4116B), 0.5 ml hEDF (cc-4317B), 0.5 ml GA (cc.4381B). Cells were cultured in a humidified incubator at 37°C and 5% CO_2_. HMVEC were used at passage 4 and 5. The flow experiments were performed using an IBIDI Unidirectional Laminar Flow pump system (IBIDI, Martinsried, Germany). IBIDI slides (0.4 mm, IbiTreat, 80176) were coated with 75,000 cells per slide and incubated for 24h in static condition to allow the cells to attach and grow to confluence. Then the flow was started at 30 ml/min (perfusion set selection 15cm, 1.6 mm; applied pressure: -15 mbar, with a flow rate of 9.8ml/min corresponding to a shear stress of 12.9 dyne/cm^2;^ next continuous switching operation 30 sec) as described earlier [20]. After 48 hours, the cells were stimulated with 100nM insulin (Actrapid, Novo Nordisk, Sweden) for 24 hours followed by direct lysis of the cells by addition of TriZol for 30 minutes at 4°C to isolate the RNA.

To measure GPIHBP1 protein expression HMVEC-ad cells were seeded at a density of ~25,000 cells per well in a 12-well plate and incubated with 100 nM insulin as described above. Cells were fixed with 4% paraformaldehyde, blocked with 10% fetal bovine serum, and incubated overnight with the goat anti-human GPIHBP1 (1:50, sc-87809, Santa Cruz). GPIHBP1 was detected with a FITC-conjugated donkey anti-goat IgG antibody (1:500, Jackson ImmunoResearch) and the nuclei were counterstained with DAPI. The images were made using Leica DMRA phase contrast microscope and images were analyzed using Image Pro Plus software. All experiments were performed in triplicate.

### Gene expression analysis

Tissue samples were homogenized in Trizol (Invitrogen) using the MagnaLyser beads followed by RNA isolation and gene expression analysis as described by the manufacturer. 1μg of mRNA was converted by reverse transcription into cDNA using iScript cDNA synthesis kit (BioRad, Uppsala, Sweden). Real time quantitative PCR (RT-PCR) was performed with 15 ng cDNA, 1μM specific primer pairs and 5μl SensiMix SYBRgreen (Bioline, UK) in a total volume of 10 μl using the CFX 384 system (BioRad) as follows: initial denaturation at 95°C for 10 min, followed by 42 cycles of 30s at 95°C, 1 min at 55°C for annealing and 40 secs at 72°C for elongation. The amplification was ended with a melting curve starting after 40 cycles. Gene expression was calculated using acidic ribosomal phosphoprotein P0 (*ARPP0 or 36B4*) as a housekeeping gene. Relative gene expression was calculated using 2 ^ΔΔCt^. Intron-exon spanning primer sequences were designed using the Primer3 program and are listed in S1 Table.

### Statistical analysis

Results are expressed as mean ± SD. Student’s T-test statistics or non-parametric Mann-Whitney test were performed to compare the groups as indicated in each separate experiment. The nonparametric Spearman Rank Correlation coefficient (r) test was performed to determine the correlation coefficients. All statistical analysis was performed using IBM SPSS Statistics (v24). A value of P<0.05 was considered statistically significant. All graphics were prepared using the GraphPrism software.

## Results

### Effect of insulin on GPIHBP1 expression in human microvascular endothelial cells

**Fig 1:**
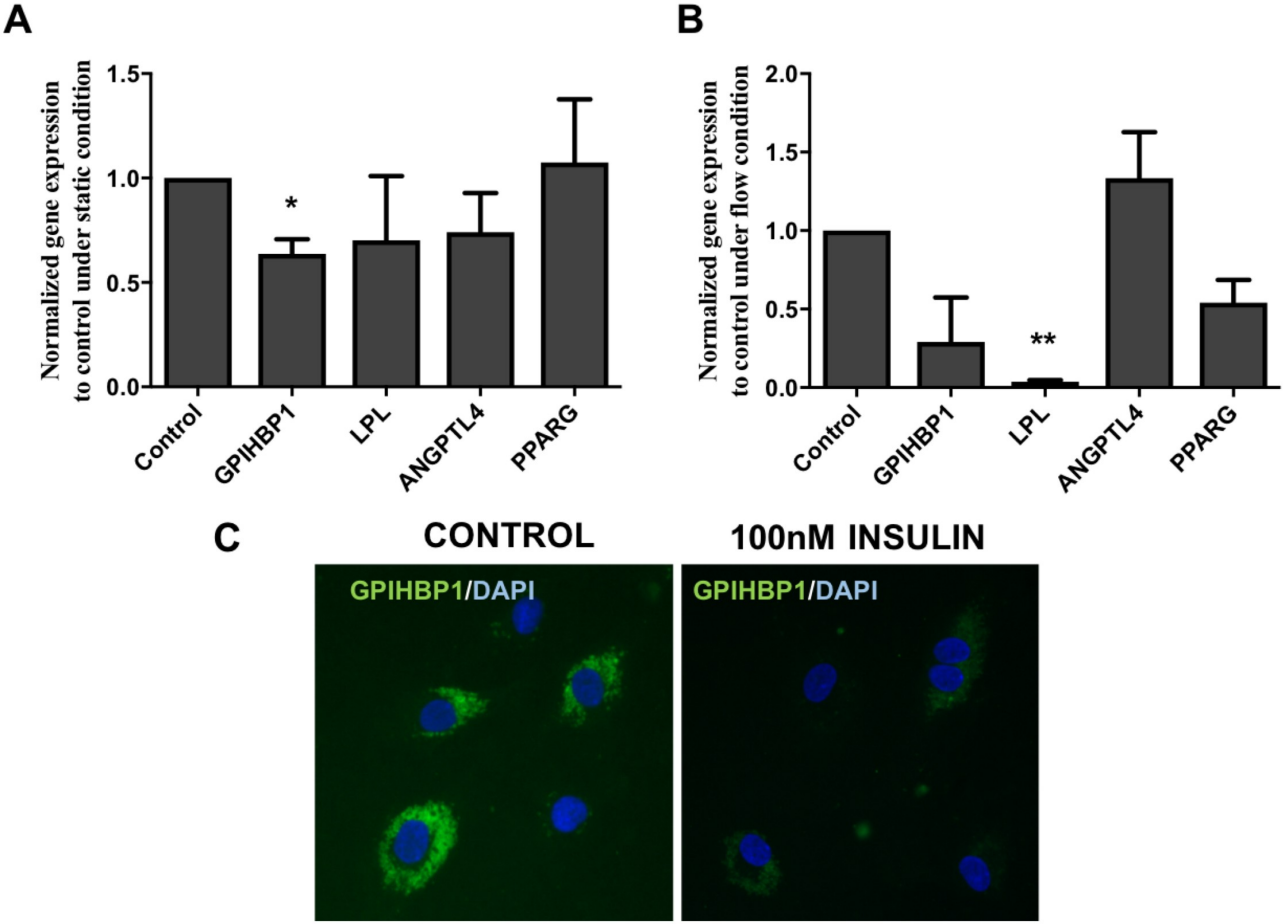
Effect of insulin on *GPIHBP1* mRNA and protein expression in HMVEC under static and flow conditions. HMVECs (p4-p5) were cultured with or without 100nM insulin for 24 hours under static (A) or laminar flow conditions (B) as described. Gene expressions of *GPIHBP1*, *LPL*, *ANGPTL4* and *PPARG* were analysed in HMVEC under static (Fig 1A) and under flow condition (Fig 1B) were measured. Data are from 4 independent experiments and presented as mean ± SD. Representative immunofluorescence image of HMVECs stained for GPIHBP1 protein under static condition. Differences were analysed using Mann-Whitney nonparametric test. *P<0.05 and **P<0.01.

To determine if insulin affects *GPIHBP1* mRNA and protein expression, HMVECs were stimulated with a high concentration of insulin under static (Fig 1A) as well as flow conditions (Fig 1B), a model which will make the cells insulin resistant. Exposure to insulin resulted in a significant 70% decrease in *GPIHBP1* mRNA expression compared to the non-stimulated cells under static conditions (Fig 1A; p<0.05). Under flow conditions, a decrease in both LPL(p<0.001) and GPIHBP1 mRNA expression was found (Fig 1B), whereas the difference in *PPARG* mRNA expression, which is a known regulator of GPIHBP1 mRNA [21], did not reach statistical difference. Of note, *ANGPTL4* expression remained unchanged. This was accompanied by reduced immunofluorescence staining for GPIHBP1 protein in insulin stimulated cells under static condition as compared to the control cells (Fig 1C).

### Decreased GPIHBP1 protein levels in Lepr ^db/db^ mouse model

**Fig 2:**
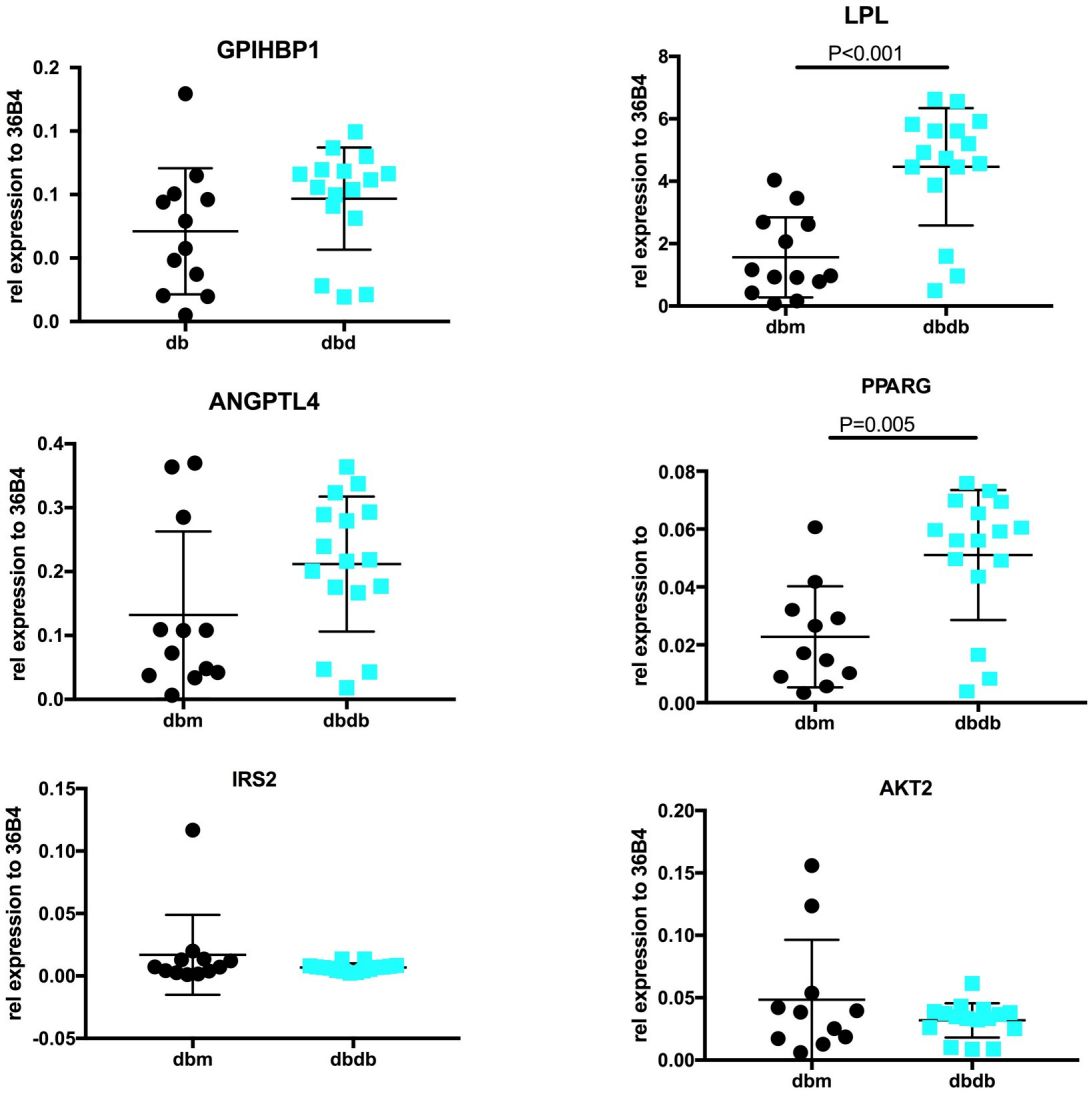
mRNA expression of genes involved in peripheral lipolysis in visceral adipose tissue lysates from *Lepr*^*db/m*^ and *Lepr*^*db/db*^ mice. Visceral adipose tissue was harvested from *Lepr*^*db/m*^ and *Lepr*^*db/db*^ mice (n = 8 per group). A. mRNA expression of *Gpihbp1*, *Lpl*,*Angptl4*, *Pparg*, *Irs2* and *Akt2*. Relative expression was determined using *36B4* as the housekeeping gene.

**Fig 3:**
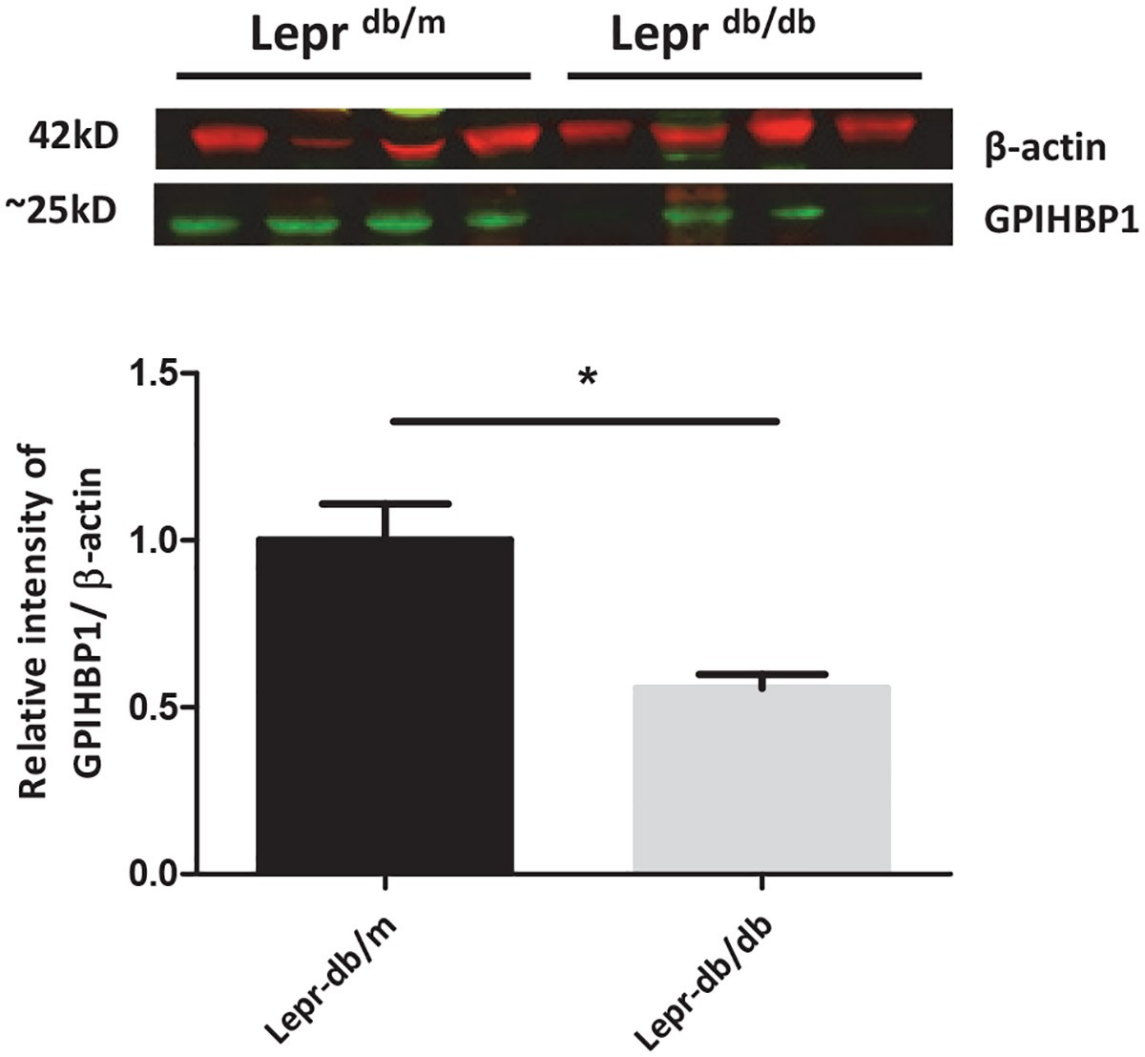
Representative blots for GPIHBP1 protein expression in visceral adipose tissue of *Lepr*^*db/m*^ and *Lepr*^*db/db*^ mice. Results are expressed as means ± SD; t-test statistics were used to test for differences between the groups. *P<0.05.

To further explore the role of insulin in peripheral lipolysis, we studied *Gpihbp1* mRNA and protein expression in a well-established type 2 diabetic mouse model, the insulin resistant *Lepr*^*db/db*^ and control *Lepr*^*db/m*^ mice. *Lepr*^*db/db*^ mice, on a chow diet, are obese, have significantly increased plasma insulin levels (18.9 ± 1.6 pmol/ vs 1.4 ± 0.6 pmol/l), increased plasma TG levels (0.75 ± 0.6 mmol/l vs 0.56 ± 0.2 mmol/l resp.) as well as increased plasma cholesterol levels reflecting an insulin resistant state (4.57 ± 0.8 mmol/l vs 1.87 ± 0.15 mmol/l; P<0.001; Table 1) as compared to control *Lepr*^*db/m*^ mice. Since visceral adipose tissue is the major organ involved in Gpihbp1-mediated lipolysis, gene expression of lipolysis associated genes were assessed in visceral adipose tissue depots. *Lpl* and *Pparg* mRNA expressions were significantly increased in *Lepr*^*db/db*^ mice (P<0.001 and P<0.005 resp.; Fig 2). *Gpihbp1* mRNA expression was not different, whereas *Angptl4* mRNA expression was slightly but not significantly increased. Interestingly, both *Irs2* and *Akt2* mRNA expression were unchanged in *Lepr*^*db/db*^ as compared to control *Lepr*^*db/m*^ mice. Although *Gpihbp1* mRNA expression in the visceral adipose tissue was not different between the mouse models, Gpihbp1 protein levels in visceral adipose tissue were significantly lower in *Lepr*^*db/db*^ mice as compared to *Lepr*^*db/m*^ mice. (P<0.05, Fig 3).

**Table 1:** Biochemical characteristics of *Lepr*^*db/db*^ and *Lepr*^*db/m*^ mice.

Parameter	*Lepr*^*db/m*^	*Lepr*^*db/db*^
N	11	16
Body weight (g)	30.6 ± 03	45.9 ± 1.1 *
Insulin (pmol/l)	1.4 ± 0.6	18.9 ± 1.6*
Leptin (ng/ml)	5.68 ± 2.0	>60
Cholesterol (mmol/l)	1.87 ± 0.2	4.57 ± 0.8
Triglycerides (mmol/l)	0.59 ± 0.2	0.75 ± 0.6

Data are expressed as mean ± SD.

* indicates p<0.05 between *Lepr*^*db/m*^ and *Lepr*^*db/db*^ mice using Student T-test statistics.

**Fig 4:**
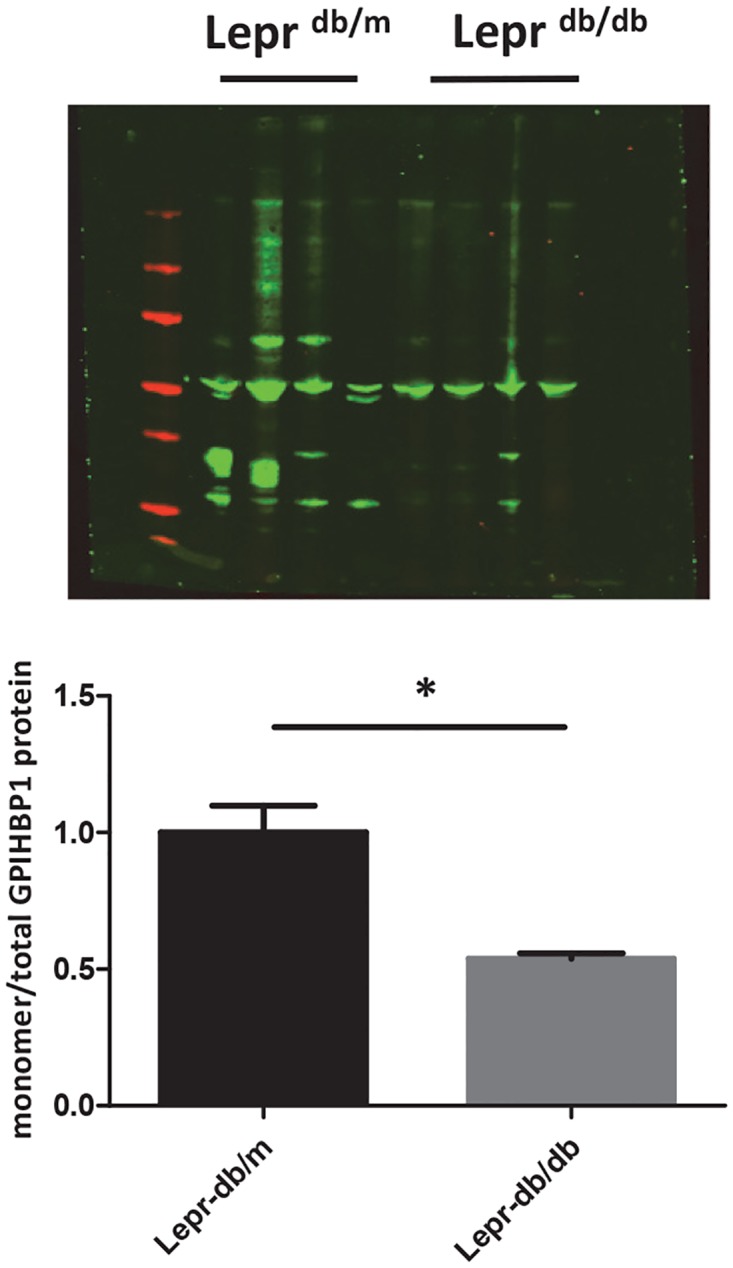
Detection of monomeric and multimeric forms of Gpihbp1 in visceral adipose lysates from *Lepr*^*db/m*^ and *Lepr*^*db/db*^ mice. GPIHBP1 protein was separated on SDS-PAGE as described using non-reducing conditions. GPIHBP1 was visualized as described. Data are expressed as mean ± SD. * P<0.05.

It has been shown that only GPIHBP1 protein in the form of a monomer is capable of binding LPL [22]. To elucidate whether Gpihbp1 folding is impaired in the diabetic mice, Gpihbp1 monomer/multimer ratio was assessed in tissue lysates derived from visceral adipose tissue of both *Lepr*^*db/db*^ and *Lepr*^*db/m*^ mice. Indeed, a significant reduction in the monomeric form of Gpihbp1 was observed in *Lepr*^*db/db*^ mice (Fig 4; P<0.01), which is suggestive for a reduced capacity in visceral adipose tissue to lipolyse TG in *Lepr*^*db/db*^ mice.

### Association between plasma insulin levels and GPIHBP1 protein in human visceral adipose tissue

**Fig 5:**
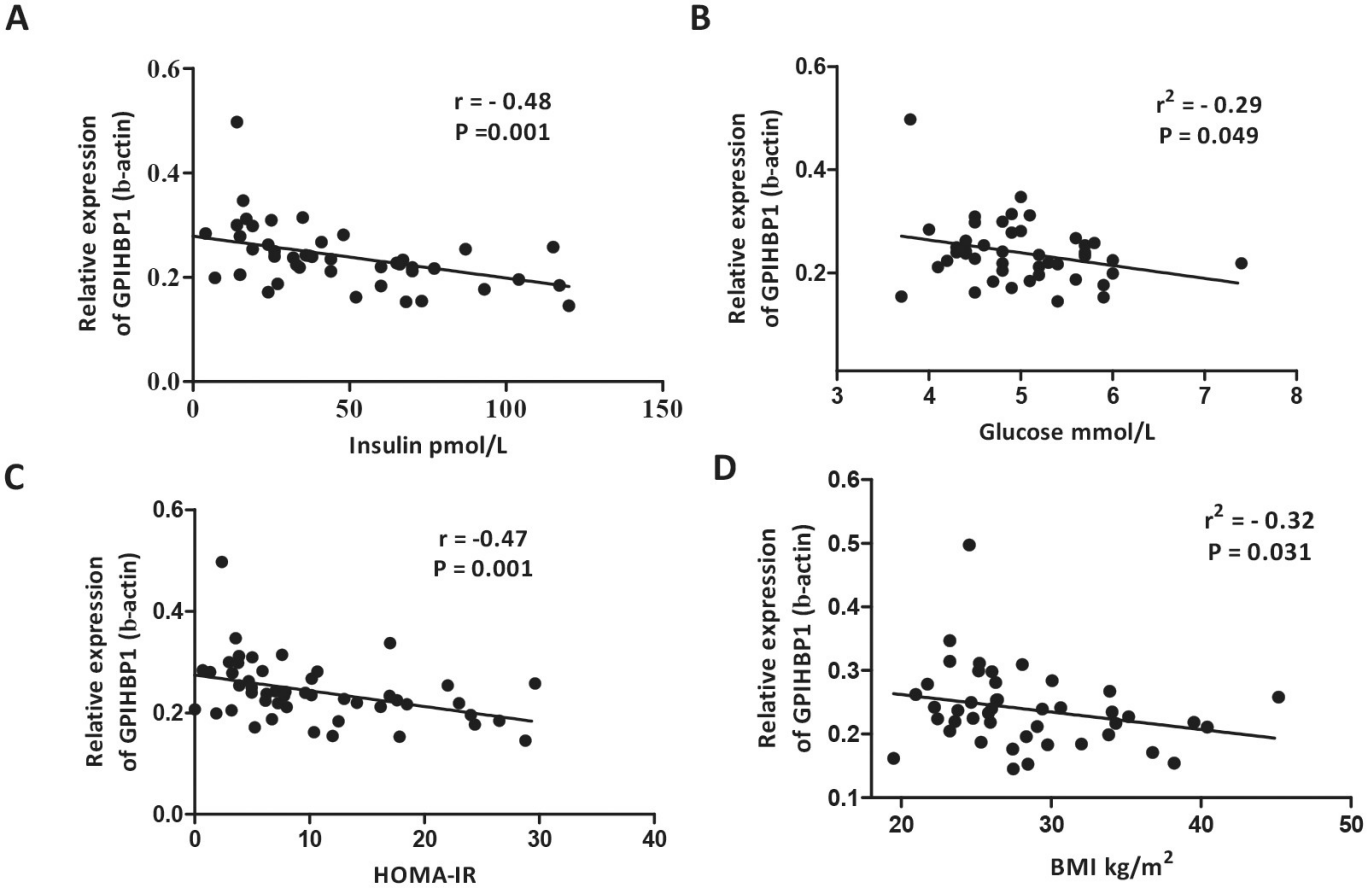
Correlation between visceral adipose tissue GPIHBP1 protein levels and plasma insulin, plasma glucose, HOMA-IR and BMI. GPIHBP1 protein levels in visceral adipose tissue biopsy specimen were measured in 45 subjects A. correlation with plasma insulin levels, B. plasma glucose levels, C. HOMA-IR and D. BMI. Correlations were tested by Pearson’s correlation coefficient test. BMI, body mass index, HOMA-IR, Homeostasis Assessment of Insulin resistance. HOMA-IR assessed using the formula: glucose [(mmol/L)*insulin (μIU/L)]/22.5.

Finally, to investigate the clinical relevance of GPIHBP1 protein expression in human visceral adipose, we evaluated *GPIHBP1* mRNA and protein levels in visceral adipose tissue biopsies obtained from humans participating in a small clinical study. The baseline biochemical characteristics are displayed in Table 2. The participants were lean to moderately obese with an average BMI of 28.4 ± 5.6 kg/m^2^. Plasma TG levels were 1.2 mmol/l (range: 0.3–3.1 mmol/L) and HOMA-IR levels 1.54 ± 1.13 with a range from 0.1–4.2. Next, we analyzed *GPIHBP1*, *LPL*, *ANGPTL4 and PPARG* mRNA expression in visceral adipose tissue. No significant correlation was found between gene expression analysis and metabolic biochemical parameters except for a significant correlation between *ANGPTL4* expression and plasma TG levels (r = 0.357; p<0.05). *GPIHBP1* gene expression was, however, significantly correlated with mRNA expression of *LPL* (r = 0.702; P<0.01), *ANGPTL4* (r = 0.547; P<0.01) and *PPARG* (R = 0.535; P<0.01), replicating the findings of Davies etal who showed a similar relationship in mice.[21] Since *GPIHBP1* mRNA expression may not reflect the physiological state, we additionally analysed GPIHBP1 protein in visceral adipose tissue biopsy material. We found that GPIHBP1 protein levels were significantly correlated with plasma insulin levels (r = -0.49 p = 0.001; Fig 5A), plasma glucose levels (r = -0.29, p = 0.049; Fig 5B), HOMA-IR (r = -0.47, p = 0.001; Fig 5C) and BMI (r = -0.32, p = 0.031; Fig 5D). To assess the contribution of BMI, insulin, glucose and HOMA-IR to the variability in the GPIHBP1 protein level, we performed a multiple regression analysis using these 4 parameters in one model. Insulin, glucose and HOMA-IR remained significantly associated with GPIHBP1 protein levels indicating that these variables explain up to 37% of the variability of GPIHBP1 protein levels (Table 3).

**Table 2:** Anthropometric and biochemical characteristics of the subjects.

Parameter	
N	45
Male (%,N)	68.9 (31)
Age (years)	46 ± 1
BMI (kg/m^2^)	28.4 ± 5.6
Glucose (mmol/l)	5.0 ± 0.7
Insulin (pmol/l)	46.6 ± 31.0 [4–120]
HOMA-IR	1.54 ± 1.13 [0.1–4.2]
HbA1C (%)	5.5 ± 0.3
Cholesterol (mmol/l)	5.2 ± 0.8
LDLc (mmol/l)	3.2 ± 1.0
HDLc (mmol/l)	1.4 ± 0.4
TG (mmol/l)	1.2 [0.3–3.1)

Data are presented as mean ± SD or as median [minimum-maximum]. BMI, body mass index; HOMA-IR, Homeostasis Assessment of Insulin resistance; TG, triglycerides. LDLc = LDL cholesterol; HDLc = HDL cholesterol; HOMA-IR assessed using the formula: glucose [(mmol/L)*insulin (μIU/L)]/22.5.

**Table 3:** Multiple linear regression for GPIHBP1 protein expression in visceral adipose tissue.

	GPIHBP1 protein expression in visceral adipose tissue	
Parameter	β—coefficient	p value
BMI	- 0.034	0.797
Insulin	- 4.348	0.000
Glucose	- 0.935	0.002
HOMA-IR	4.397	0.001
TG	- 0.149	0.267

Analysis were performed using a regression model with these 4 parameters that were significantly correlated with GPIHBP1 protein as shown in Fig 4.

## Discussion

The current study demonstrates that visceral adipose tissue GPIHBP1 protein concentration (rather than gene expression) in visceral adipose tissue is affected in a chow-fed murine model of insulin resistance, the *Lepr*^*db/db*^ mice. In human visceral adipose tissue we observed a correlation with plasma insulin levels, suggesting that peripheral lipolysis capacity may be impaired. This intriguing observation may partly underlie the increased (postprandial) plasma TG levels observed in atherogenic dyslipidemic phenotype.

It has been generally accepted that insulin resistance is associated with the presence of the atherogenic dyslipidemia, which is defined by the presence of moderate elevated plasma TG levels and reduced HDL cholesterol levels [2]. In this regard, insulin plays an important role in the metabolism of apoB-containing TG rich particles. Insulin regulates apoB homeostasis in the liver by increasing apoB degradation and thereby reducing hepatic VLDL secretion [23]. Thus, in case of insulin resistance, this control mechanism is impaired and as a consequence apoB degradation is suppressed leading to enhanced secretion of apoB-containing VLDL particles from the liver [24, 25]. An important determinant for TRL clearance is LPL-mediated lipolysis prior to hepatic receptor-mediated clearance [8]. LPL-mediated lipolysis is the first rate-limiting step in TG metabolism and has been shown to be regulated by insulin. GPIHBP1, expressed at the endothelial cell surface in small capillaries, is essential for LPL-mediated lipolysis to occur. The task for LPL is thus to redirect fatty acids to adipose tissue for energy storage. LPL is synthesized and secreted by adipocytes and muscle which is partly regulated by insulin both post-transcriptionally and post-translationally [26]. In an insulin-resistant state this regulation is lost.[26] Newly synthesized LPL has to be transported towards the endothelial cell surface where it can perform its hydrolytic action. Recently, GPIHBP1 has been recognized as the protein on the endothelial cell surface that binds LPL and facilitates TG lipolysis in the peripheral tissues (adipose tissue, heart and skeletal muscle).[27] Loss of function mutations in *GPIHBP1* result in impaired lipolysis capacity leading to severe hypertriglyceridemia [17, 28–30]. Very little is known about the regulation of GPIHBP1 in the setting of metabolic abnormalities including the presence of insulin resistance. Kroupa and co-workers showed a direct effect of insulin on *Lpl*, *Gpihbp1* and *Angptl4* adipose tissue mRNA expression in rats 60 minutes after a direct injection of insulin in the adipose tissue [31], revealing that insulin directly affected *Gpihbp1* and *Angptl 4* expression. However, the lower Angptl4 expression rescue LPL which remains active due to sufficient Gpihbp1. In line with this study, our results show that *GPIHBP1* mRNA levels significantly decreased when human microvascular endothelial cells were stimulated with high concentrations of insulin, creating an insulin resistant state, which coincided with a reduction in GPIHBP1 protein expression. *LPL* mRNA expression also decreased whereas *ANGPTL4* mRNA expression was unchanged, suggesting a direct effect of insulin on gene and protein expression mediated by insulin in these *in vitro* experiments. We additionally show that in chow-fed *Lepr*^*db/db*^ mice, who are in an insulin resistant state, *Gpihbp1* mRNA expression in visceral adipose tissue was unchanged but *Lpl* mRNA expression was significantly increased which is comparable to observations in an earlier study in rats [31]. Increased information was obtained by measuring Gpihbp1 protein expression which was significantly reduced in *Lepr*^*db/db*^ mice. This strong reduction in Gpihbp1 protein may have consequences for the lipolytic capacity in visceral adipose tissue phenotype in insulin resistant state. Indeed, *Gpihbp1*^*-/-*^ mice lack the capacity to properly hydrolyze TG which turns out to be responsible for metabolic perturbations in adipose tissue [32]. Recent data from Beigneux etal [22] suggested that the monomeric form of GPIHBP1 protein is the active form which avidly binds LPL. We thus asked the question whether insulin resistance may influence the formation of dimer/multimer GPIHBP1 which ultimately leads to reduced lipolytic capacity in adipose tissue. Indeed, as shown in Fig 4, a reduction in the monomeric active form of Gpihbp1 was found in visceral adipose tissue material obtained from *Lepr*^*db/db*^ mice. So, on top of a reduction in total protein levels we additionally observed a reduction in active Gpihbp1. It has been shown that GPIHBP1 is N-glycosylated which is important for its proper function[33]. Whether changes in N- glycosylation may underlie the behavior of GPIHBP1 in an insulin resistant state has to further investigated as the actual mechanism involved in multimerisation of Gpihbp1 is currently unknown. Future studies should be thus focused upon investigating the consequence of active/inactive GPIHBP1 under different physiological conditions, including a high fat diet which may lead to more pronounced changes in the plasma lipid phenotype as a consequence of reduced lipolytic capacity.

**Fig 6:**
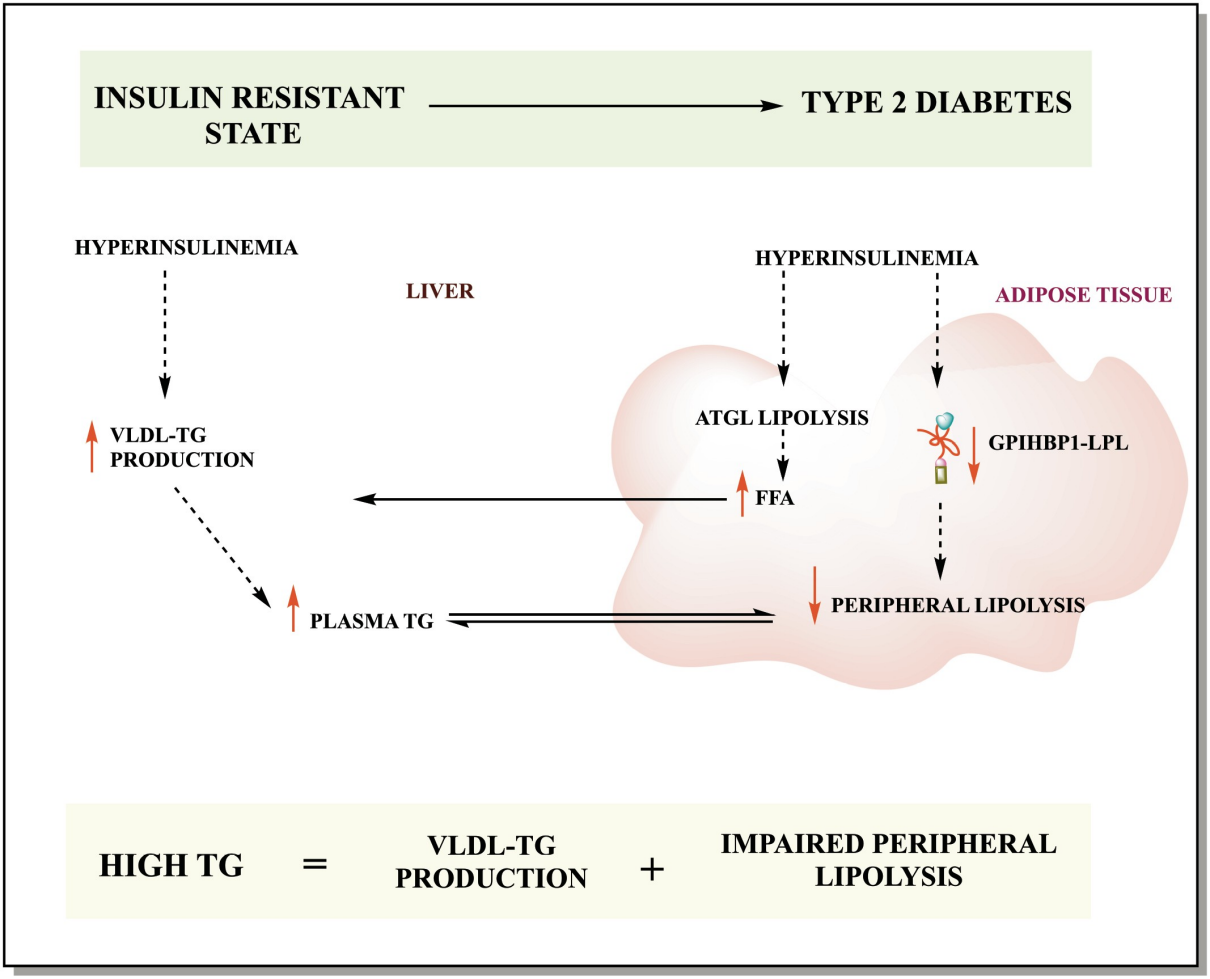
Proposed mechanism. In this illustration we show the proposed underlying mechanism of GPIHBP1 function in TG homeostasis in an insulin resistant state. Hyperinsulinemia leads to decreased protein levels of GPIHBP1 in adipose tissue that results in decreased LPL-mediated peripheral lipolysis of TG. This, in turn, leads to elevated plasma levels of TG and increased VLDL-TG increase due to hepatic insulin resistant state.

The atherogenic dyslipidemia in obesity and type 2 diabetes, that has been described extensively in the literature, has been attributed to impaired hepatic VLDL secretion as well as diminished hepatic remnant uptake. However, data on the effect of impaired peripheral lipolysis capacity are lacking. Ruge etal [34] was unable to demonstrate an association subcutaneous adipose tissue *GPIHBP1* mRNA expression and metabolic parameters. Our data in visceral adipose tissue again could not demonstrate an association between *GPIHBP1* mRNA expression and metabolic parameters. However, in the present study, protein levels of GPIHBP1 in visceral adipose tissue were found to be negatively correlated with plasma insulin level, plasma glucose level and HOMA-IR. This suggests that in visceral adipose tissue, GPIHBP1 is mainly post-translationally modulated. This regulation is impaired in an insulin resistant state. Unfortunately, in the human study we were unable to test for GPIHBP1 monomer/multimer due to lack of sufficient tissue sample, which is a limitation of this study. We also have to stress that our subject population only has a mild insulin resistance phenotype. But even in this mild condition we already observe the negative changes that eventually will lead to the development of the atherogenic dyslipidemia observed in T2DM patients. In Fig 6 we propose a graphical view of the metabolic changes that occur in adipose tissue in an insulin resistant state.

In summary, we show, using 3 different models that insulin is involved in the regulation of GPIHBP1 in visceral adipose tissue. We show that GPIHBP1 protein expression is decreased as is the amount of active monomeric Gpihbp1, leading to impaired peripheral lipolysis capacity. Our data thus underscore the importance of measuring GPIHBP1 protein instead of only mRNA to fully elucidate the mechanism involved in future studies.

## Supporting information

S1 Table Primer sequences for RT-PCR anal. (DOCX)

S1 File Image plosone (1).pdf. (PDF)
