# Supplementary material for: Decreased GPIHBP1 protein levels in visceral adipose tissue partly underlie the hypertriglyceridemic phenotype in insulin resistance
Source: PLoS One. 2018 Nov 8;13(11):e0205858. doi: 10.1371/journal.pone.0205858 (PMC6224034; doi:10.1371/journal.pone.0205858)
Supplement: S1 Table — (DOCX) [file pone.0205858.s001.docx]

**SUPPLEMENT MATERIALS**

Table S1 RT-PCR Primer sequences

| Genes | Forward | Reverse |
| --- | --- | --- |
| **Human**  36B4(ARPPO)  GPIHBP1  LPL  ANGPTL4  PPARG  **Mice**  36B4(Arppo)  Gpihbp1  Lpl  Angptl4  Pparg  Irs2  Akt2  Fasn  Dgat2  Scd1  Acaca  Acacb  Elovl6  Cpt1a | ACGGGTACAAACGAGTCCTG  CTGCAACCTGACGCAGAAC  ATGTGGCCCGGTTTATCA  CAACCTCAACGGCCAGTACTTC  GACAGGAAAGACAACAGACAAATC  GGACCCGAGAAGACCTCCTT  ACCAACATGATCCCTGGAAG  CTCGCTCTCAGATGCCCTAC  GTTTGCAGACTCAGCTCAAGG  TGAGCACTTCACAAGAAATTACCA  ACAACCTATCGTGGCACCTC  TTTGCACTCGAGAGATGTGG  GCTGCTGTTGGAAGTCAGC  ATCCTTCCTGGTGCTAGGAGT  TTCCCTCCTGCAAGCTCTAC  GTCCCCATCACCACTCCTTC  GAGGCCCAGAGAACACGTAG  AGAGGGGAGGACAGAGACTG | GCCTTGACCTTTTCAGCAAG  CTCCACCGTCTTGGTGATG  CTGTATCCCAAGAGATGGACATT  CCGCCAGGTCTTCCAGAA  GGGGTGATGTGTTTGAACTTG  GCACATCACTCAGAATTTCAATGG  CTGGAGCAGCTCTGTGTCTG  CCACTGTGCCGTACAGAGAA  CCAAGAGGTCTATCTGGCTCTG  GTCCACAGAGCTGATTCCGA  CCATGAGACTTAGCCGCTTC  AGTGTTCGTTCCTCGGAGTG  GCCAGCCAGGTGAAGTAGAG  CAGAGCGCTGGTCATGTAGT  CCCCTTCAGAGTTGGGTGAG  TGGATGGCTTCGTTCTCGTT  TCTGCTCTGCCGTTGTTGTG |

Primers were designed using the program Primer3. All primers are crossing the intron-exon boundaries.
