# Supplementary material for: Decreased GPIHBP1 protein levels in visceral adipose tissue partly underlie the hypertriglyceridemic phenotype in insulin resistance
Source: PLoS One. 2018 Nov 8;13(11):e0205858. doi: 10.1371/journal.pone.0205858 (PMC6224034; doi:10.1371/journal.pone.0205858)
Supplement: S1 File — (PDF) [file pone.0205858.s002.pdf]

ID	gender	age	length	weight	BMI	WHR	glucose	Hba1c
1.00	1.00	52.00	1.79	88.50	27.66	0.92	6.00	5.90
2.00	1.00	59.00	1.62	66.00	25.15	1.01	4.80	5.60
3.00	1.00	40.00	1.74	79.50	26.41	0.95	4.60	5.50
4.00	2.00	56.00	1.80	82.00	25.31	0.99	5.60	5.60
5.00	1.00	49.00	1.69	72.00	25.21	0.81	5.10	5.20
6.00	1.00	38.00	1.76	72.00	23.24	0.94	4.80	5.30
8.00	2.00	42.00	1.82	72.00	21.74	0.87	4.90	5.50
10.00	2.00	56.00	1.73	88.00	29.40	1.03	5.70	5.60
12.00	1.00	30.00	1.63	105.00	39.52	0.91	7.40	6.00
13.00	1.00	34.00	1.63	120.00	45.17	0.98	5.80	5.70
14.00	1.00	40.00	1.65	110.00	40.40	0.93	4.10	5.40
15.00	1.00	53.00	1.62	61.00	23.24	0.82	5.00	5.40
16.00	2.00	48.00	1.75	86.00	28.08	#NULL!	4.50	5.60
18.00	2.00	48.00	1.76	73.00	23.57	0.90	5.30	5.90
19.00	1.00	27.00	1.76	76.00	24.54	0.91	3.80	5.50
20.00	1.00	42.00	1.65	104.00	38.20	0.95	3.70	5.80
21.00	1.00	42.00	1.56	60.00	24.65	1.02	4.30	5.40
22.00	1.00	40.00	1.69	64.00	22.41	0.83	4.20	5.00
23.00	1.00	41.00	1.73	90.00	30.07	0.78	4.00	4.90
25.00	1.00	51.00	1.59	53.00	20.96	0.87	4.40	5.40
26.00	2.00	44.00	1.76	72.00	23.24	0.92	4.90	5.20
27.00	2.00	44.00	1.93	126.00	33.83	1.06	6.00	6.10
28.00	1.00	71.00	1.55	62.00	25.81	0.88	5.70	6.10
29.00	1.00	49.00	1.69	105.00	36.76	0.89	4.90	5.50
30.00	2.00	57.00	1.78	83.50	26.35	0.94	5.70	5.70
32.00	1.00	25.00	1.63	59.00	22.21	0.78	4.40	5.10
33.00	2.00	45.00	2.02	125.00	30.63	0.86	4.80	5.30
35.00	1.00	52.00	1.68	55.00	19.49	0.88	4.50	5.20
36.00	2.00	68.00	1.80	89.00	27.47	1.09	5.40	5.90
37.00	1.00	71.00	1.67	83.00	29.76	0.86	4.70	5.40
39.00	2.00	60.00	1.83	83.00	24.78	0.96	6.00	5.60
40.00	1.00	36.00	1.68	73.50	26.04	0.83	4.50	5.50
41.00	2.00	38.00	1.80	84.00	25.93	0.92	4.80	5.30
42.00	2.00	36.00	1.75	84.00	27.43	0.92	5.90	5.10
43.00	1.00	47.00	1.60	90.00	35.16	0.89	4.50	5.90
44.00	1.00	27.00	1.80	77.00	23.77	0.86	4.40	5.10
45.00	1.00	63.00	1.70	84.00	29.07	0.85	5.20	5.30
46.00	1.00	51.00	1.58	71.00	28.44	0.97	5.90	5.90
47.00	1.00	49.00	1.61	83.00	32.02	0.94	5.10	5.60
48.00	1.00	46.00	1.62	89.00	33.91	0.92	5.60	5.60
49.00	1.00	58.00	1.72	77.00	26.03	0.88	4.30	5.30
50.00	2.00	55.00	1.86	98.00	28.33	0.98	5.20	5.20
52.00	1.00	41.00	1.69	98.00	34.31	0.95	5.40	5.40
53.00	1.00	33.00	1.67	95.00	34.06	0.85	5.20	5.20
55.00	1.00	34.00	1.70	76.00	26.30	0.83	5.00	5.10

insulin	chol	TG	HDLc	LDLc	LPLv	GPIHBP1v	PPARgv
17.00		0.60	1.50	4.30	0.28519	0.03326	0.09408
14.00	5.6	1.10	1.50	3.60	0.50348	0.21022	0.20448
19.00	7.1	1.60	1.40	5.00	1.28343	0.05555	0.18301
27.00	5	1.20	1.00	3.50	0.11663	0.01552	0.03874
17.00	4.5	1.20	1.20	2.80	1.43396	0.03420	0.09278
15.00	6.9	1.30	1.00	5.30	0.09539	0.06887	0.01734
15.00	5.5	1.70	1.30	3.10	0.09087	0.00436	0.01488
38.00	4.9	1.00	1.60	2.90	0.94606	0.05219	0.15604
70.00	5.4	3.10	0.90	4.60	1.71713	0.11034	0.28917
115.00	5.5	1.90	1.10	3.50	0.32086	0.02091	0.08304
44.00	5.4	1.60	0.90	3.80	0.31208	0.01201	0.04704
16.00	5.6	1.60	1.90	1.70	3.78423	0.08657	0.26062
25.00	5.1	1.60	0.90	3.00	0.26062	0.02401	0.04737
60.00	5.2	2.20	1.70	3.30	0.57038	0.03235	0.13774
14.00	4.8	0.50	1.30	3.30	0.89503	0.06037	0.07802
73.00	5	0.90	1.00	3.60	0.47963	0.01782	0.13584
26.00	5.8	2.10	1.00	5.10	0.12414	0.00367	0.04481
33.00	4.4	0.80	1.60	2.40	0.29937	0.01448	0.08133
4.00	3.4	0.50	1.40	1.80	0.27548	0.00955	0.09876
24.00	6.1	1.70	1.40	3.90	1.40444	0.04544	0.12158
35.00	4.7	0.80	1.50	2.90	0.85263	0.07484	0.19888
7.00		0.80	0.90	#NULL!	0.21169	0.01184	0.03019
67.00	5.1	0.50	3.00	1.90	1.04247	0.02120	0.09408
24.00	3.6	0.80	1.00	2.20	0.45376	0.02005	0.04481
87.00	5.6	1.00	1.30	3.90	0.93952	0.05995	0.12941
36.00	5.3	1.00	1.50	3.30	0.31425	0.00814	0.10224
37.00	4.6	1.40	0.90	3.10	0.27739	0.00923	0.08597
52.00	4.6	2.10	1.40	2.90	0.44751	0.03040	0.12941
120.00	5.4	1.90	1.50	3.50	0.24656	0.01234	0.11582
60.00	5.5	1.70	2.10	2.60	0.16380	0.00724	0.06561
66.00	4.1	1.00	1.00	2.70	0.20027	0.00936	0.18816
19.00	4.1	0.70	1.40	2.40	0.21464	0.00178	0.13966
34.00	5.5	0.80	2.20	2.90	0.23488	0.00867	0.08021
93.00	5.7	1.70	0.90	4.00	0.64171	0.09278	0.20877
65.00	6.4	0.90	1.70	4.30	0.20733	0.02896	0.04671
32.00	3.5	0.30	1.90	1.50	0.23165	0.01418	0.07748
70.00	4.5	0.40	1.80	2.50	0.14968	0.00443	0.01563
68.00	5.4	1.30	1.20	3.60	0.22688	0.02538	0.14865
117.00	6.3	1.40	1.50	4.20	0.40613	0.02592	0.13490
41.00	4.8	2.00	1.00	3.70	0.12500	0.02896	0.05219
26.00	6	1.40	1.50	3.90	0.73713	0.04607	0.15496
104.00	5.2	2.00	1.20	3.10	0.53961	0.02610	0.06293
77.00	5.4	0.70	1.50	3.60	0.24827	0.00873	0.06792
44.00	4.5	0.80	1.50	2.60	0.14660	0.00347	0.07330
48.00	5.3	0.70	1.90	3.10	0.05007	0.00375	0.03019

ANGPTL4_VAT	GPIHBP1protvis
0.0242122	#NULL!
0.0127016	0.2995660
#NULL!	0.2538610
0.0106407	0.1871250
0.0811556	0.3116315
0.0126690	0.2045885
0.0215311	0.2784397
0.0606340	0.2392068
0.1141154	0.2187131
0.0153378	0.2579230
0.0531279	0.2111989
0.0583013	0.3468086
0.0502379	0.3094169
0.0435033	0.2196882
0.0201158	0.4972488
0.0517364	0.1544445
0.0043885	0.2497413
0.0226420	0.2238476
0.0161650	0.2839949
0.0451701	0.2624189
0.0563881	0.3143281
0.0278594	0.1988814
0.0223741	0.2331904
0.0343798	0.1710941
0.0767456	0.2535696
0.0271137	0.2421433
0.0613578	0.2412970
0.0549908	0.1619891
0.0360974	0.1450196
0.0158047	0.1830681
0.0141544	0.2246792
0.0084932	0.2980904
0.0104367	0.2187434
0.0461978	0.1765194
0.0383632	0.2274929
0.0189323	0.2374367
0.0235501	0.2118989
0.0076559	0.1528429
0.0301666	0.1842906
0.0308792	0.2672546
0.0276757	0.2396337
0.0620089	0.1959645
0.0107559	0.2168196
0.0067820	0.2348068
0.0067417	0.2812324
